# Mitogenomic Characterization of *Microhyla fissipes* and Its Implications for Phylogenetic Analysis in Microhylidae

**DOI:** 10.3390/biology14101342

**Published:** 2025-10-01

**Authors:** Siqi Shan, Simin Chen, Chengmin Li, Huiling Huang, Yaqing Liao, Lichun Jiang

**Affiliations:** School of Biological and Pharmaceutical Sciences, Mianyang Teachers’ College, Mianyang 621000, China; 19839412383@163.com (S.S.); 17709049484@163.com (S.C.); 18380527400@163.com (C.L.); m18227952859@163.com (H.H.); 17873081786@163.com (Y.L.)

**Keywords:** *Microhyla fissipes*, Microhylidae, mitochondrial genome, phylogenetic relationships, genomic architecture, selection pressure

## Abstract

**Simple Summary:**

*Microhyla fissipes*, a species within the family Microhylidae, is distributed exclusively in Asia. This frog holds significant scientific and ecological importance, but research on this species remains limited to date. In this study, we report the characteristics of the mitochondrial genome of *M. fissipes* and, using these data, investigate the phylogenetic relationships within the family Microhylidae. The results indicate that the mitogenomic size of *M. fissipes* is 16,723 bp, arranged in a circular configuration, containing 13 protein-coding genes (PCGs), 22 tRNAs, 2 rRNAs, and 1 non-coding region. The nucleotide composition is 28.9% adenine (A), 31.01% thymine (T), 25.51% cytosine (C), and 14.58% guanine (G). Phylogenetic analysis indicated that Microhylidae can be divided into four monophyletic genera (*Microhyla*, *Glyphoglossus*, *Kaloula*, *Anilany*), with *M. fissipes* being most closely related to *Microhyla heymonsi*. Analysis indicates that the selection pressure ratios for most genes are less than 1, suggesting that these genes have undergone strong purifying selection. However, in the *COX2* and *COX3* genes, the Ka/Ks ratios between some species exceed 1, indicating that the evolutionary process aims to eliminate harmful mutations. This study enriches the basic biological data for *M. fissipes* and provides a fundamental reference for the conservation of *M. fissipes* and *Microhyla.*

**Abstract:**

The microhylid frog *Microhyla fissipes* is a protected terrestrial wildlife species in China, recognized for its ecological, economic, and scientific value. However, its mitochondrial genome remains poorly characterized. To address this gap, we sequenced and annotated the complete mitogenome of *M. fissipes* to elucidate its structural organization and phylogenetic placement within Microhylidae. The assembled mitogenome is 16,723 bp in length and contains 37 genes, including 13 protein-coding genes, 2 rRNAs, and 22 tRNAs, along with one control region and the origin of heavy-strand replication. We also identified eight overlapping regions and eleven intergenic spacers. The overall base composition showed an A + T bias (59.91%) with negative AT-skew (−0.04) and GC-skew (−0.27). All tRNAs displayed typical cloverleaf secondary structures, except for trnS1, which lacked the D-arm. Phylogenetic reconstruction using both maximum likelihood and Bayesian inference strongly supported the monophyly of Microhylidae and revealed a sister-group relationship between *Microhyla* and *Kaloula*. Within *Microhyla*, *M. fissipes* was most closely related to *M. heymonsi*, with which it formed a well-supported clade that also included *Microhyla okinavensis*, *Microhyla mixtura*, and *Microhyla beilunensis*. Selection pressure analysis on protein-coding genes indicated widespread purifying selection (Ka/Ks < 1) across most genes, except for *ATP8*, *COX2*, and *COX3*, which may be under relaxed selective constraints. These findings offer valuable genomic resources for the conservation of *M. fissipes* and provide new insights into the phylogeny and evolution of microhylid frogs.

## 1. Introduction

The *Microhyla fissipes* is a small-sized frog (less than 30 mm) widespread in East Asia, inhabiting environments such as water puddles, depressions, and grassy areas; the species belongs to the family Microhylidae of the order Anura [1,2]. It is distributed in diverse habitats from subtropical basins (such as Sichuan) to islands (such as Hainan), making it an ideal subject for studying how climatic and geographical factors drive the evolution of amphibian mitochondrial genomes, as well as an important model for understanding the evolutionary dynamics of the family Microhylidae. This frog is easy to collect and rear, with a fast reproduction rate, making it an important experimental subject for molecular biology and ecological research. Its phylogenetic position also provides a unique opportunity to elucidate the ancestral differentiation patterns of *Microhyla*.

As transitional organisms bridging aquatic and terrestrial life forms, Amphibia hold significant status in evolutionary biology research. With the continuous deterioration of ecological environments and reduction in habitats, amphibian populations across species are confronting substantial survival pressures, even approaching extinction [3]. Due to their slower metabolic rates compared to endotherms and their hibernation habits, frogs generally exhibit a slower molecular evolutionary rate than mammals and birds [4]. The compact and highly conserved mitochondrial genome of *M. fissipes* provides a window into the adaptive evolution of amphibians between aquatic and terrestrial habitats. Mitochondrial genes, increasingly utilized as novel molecular markers in phylogenetic studies, have garnered increasing attention and application in evolutionary analyses owing to their advantageous characteristics including small molecular size, lack of introns, and maternal inheritance pattern [5].

The family Microhylidae includes *Kaloula*, *Microhyla*, *Trichobatrachus*, *Hyperolius*, *Breviceps*, and *Hemissus*, and their phylogenetic relationships are in good agreement with traditional taxonomy [6]. Recent advances have significantly enhanced our knowledge of *Microhyla* species diversity, yet reconstructing the evolutionary origins of this genus continues to present considerable scientific challenges [7]. And *Kaloula* and *Microhyla* each form a monophyletic group that is the sister group of the branch, indicating close affinities. *Kaloula rugifera*, *Kaloula borealis*, and *Kaloula pulchra* form the sister branch of the genus *Microhyla* [8]. In the phylogenetic tree studied by Zhao et al. [9], *M. mixtura* and *M. okinavensis* belonged to the same category of the genus *Microhyla*, and the two were presented in a topological pattern of neighboring branches, sharing relatively proximate nodes. In another phylogenetic analysis by Khatiwada et al. [10], it was observed that *M. okinavensis* and *M. fissipes* present a topological pattern of adjacent branches. It is evident that the phylogenetic relationships among *M. okinavensis*, *M. fissipes*, and *M. mixtura* remain unclear, highlighting the ongoing uncertainty in the evolutionary history of this genus. A high-quality mitochondrial genome of *M. fissipes* could provide reliable molecular markers to test hypotheses of convergent evolution or incomplete lineage sorting, thereby resolving these discrepancies. Despite extensive research on *Microhyla*, studies on the mitochondrial genome of *M. fissipes* remain notably lacking—a gap that hinders our understanding of its evolutionary history and taxonomy.

Previous studies on *M. fissipes* were largely confined to limited phylogenetic analyses—only conducting simple result analyses and involving a narrow range of taxa for tree construction—with overall taxonomic coverage remaining restricted [1,2]. In contrast, this research significantly expands the investigative scope and increases its analytical depth: while prior studies only put forward preliminary phylogenetic placements for this species, our work includes species from over ten families within the order Anura to perform a thorough phylogenetic reconstruction, which resolves previously unclear evolutionary relationships. Furthermore, we present the first estimates of divergence times for these groups, offering new insights into their evolutionary timeline. Additionally, we analyze selective pressures within *Microhyla* to identify functional constraints on the mitochondrial genome—efforts that not only refine *M. fissipes*’ phylogenetic framework but also provide novel evolutionary perspectives absent from previous publications.

Building on the identified gaps and our expanded analytical approach, we amplified *M. fissipes’* complete mitogenome sequence, systematically analyzed its structural features, and further resolved the taxonomic status of this species and its close relatives. These actions not only fill the gap in comprehensive mitochondrial genome data for this group (as highlighted earlier) but also provide experimental evidence for future conservation genetics research on Microhyla species—laying a solid foundation for subsequent studies on their evolutionary trajectories and protective strategies.

## 2. Materials and Methods

### 2.1. Ethics Approval

All experimental procedures involving *M. fissipes* were performed in strict compliance with the Wildlife Protection Law of the People’s Republic of China and approved by the Scientific Research Ethics Committee of Mianyang Teachers’ College (Approval No. MSL202522). The study adhered to recognized animal welfare guidelines, and all efforts were made to minimize harm and discomfort to the animals throughout the research process.

### 2.2. Sample Collection and DNA Extraction

The samples of *M. fissipes* were collected in Mojia Town, Fucheng District, Mianyang City, Sichuan Province, Southwestern China (105.8′24.14″, 31.58′19.35″; 555 m above sea level). The interdigital webbing was sterilized with alcohol, after which approximately 25 mg of tissue was carefully excised. The area was then disinfected again prior to releasing the animal back into its natural habitat. Immediately following collection, tissue specimens were preserved under aseptic conditions in anhydrous ethanol to ensure structural and genetic integrity. All samples were stored at −20 °C. Total genomic DNA was extracted using a standard phenol/chloroform method [11]; its integrity was then determined by electrophoresis on a 0.9% agarose gel. The concentration of the extracted DNA samples was measured by spectrophotometry and the DNA was amplified by PCR.

### 2.3. PCR Amplification and Sequencing

Twelve overlapping fragments covering the entire mitogenome, each overlapping 200–350 bp, were obtained by PCR amplification and LA-PCR. PCR amplification was performed in a reaction volume of 25 µL, which consisted of 2.5 µL of 10× loading buffer, 2.0 µL of MgCl_2_ (2.5 mol/L), 1.5 µL of dNTP mixture (2.5 mM/L each), 1.0 µL of each primer pair (10 µmol/L), 1.0 µL of DNA template (20 ng/µL), 1.0 µL of each primer pair (10 µmol/L), 1.0 µL of DNA template (20 ng/µL), 0.6 µL of LA Taq polymerase (5 U/µL), and sterilized water. The PCR cycling procedure was as follows: pre-denaturation at 95 °C for 5 min, denaturation at 94 °C for 40 s, annealing at 48–62 °C for 45 s, extension at 72 °C for 1 min for 33 cycles, and extension at 72 °C for 10 min. The PCR products were detected by agarose gel electrophoresis to confirm the correct results, and then sequenced by Sanger. Finally, the mitochondrial sequence was obtained and registered in GenBank, and the accession number was PV 083740.

### 2.4. Mitogenome Assembly, Annotation and Sequence Analysis

Sequencing results were assembled, annotated and edited manually. The positions of protein-coding genes (PCGs), tRNAs, rRNA genes and D-loop regions were annotated by BLAST v1.8.4 comparison (https://blast.ncbi.nlm.nih.gov, accessed on 21 May 2025) to search for homologous sequences, which were then used as reference sequences. We constructed a mitogenome map by Proksee [12] (https://proksee.ca/, accessed on 25 May 2025) and searched for homologous sequences by tRNAscanSE [13] (https://trna.ucsc.edu/tRNAscan-SE/index.html, accessed on 26 May 2025) analyzed tRNA secondary structures using vertebrate mitochondrial genetic code sources in default search mode. With R2DT [14] (https://r2dt.bio/, accessed on 29 May 2025), we predicted the secondary structure of 12S rRNA, 16S rRNA. Secondary validation was also performed for tRNA genes using ARWEN 1.2.3 [15] software to ensure the correct structure. The MEGA 11.0 [16] software was employed to calculate the relative codon usage frequency (RSCU) and the individual base content. The AT-skews and GC-skews were calculated using the following formula AT-skew = (A − T)/(A + T) and GC-skew = (G − C)/(G + C) [17]. The overlapping regions between genes and gene spacing were calculated manually.

### 2.5. Phylogenetic Analysis

In order to explore the phylogenetic position of *M. fissipes* within its family, we selected 30 representative species covering major genera of Microhylidae based on taxonomic representativeness, geographic distribution, and data completeness. The phylogenetic trees were constructed using PhyloSuite [18] with 13 protein-coding genes, with *Bufotes pewzowi* and *Bufo japonicus* set as outgroups due to their close but distinct phylogenetic relationship with the ingroup taxa. Prior to the construction of the phylogenetic tree, multiple sequence comparisons were performed using MAFFT [19], and low-quality comparison sites in the file were trimmed using trimAl [20]. Phylogenetic trees were constructed by concatenating the trimmed PCG sequences the optimal nucleotide substitution model was identified using ModelFinder [21]. Phylogenetic analyses were performed using both maximum likelihood (ML) and Bayesian inference (BI) approaches: ML analysis was conducted with IQ-TREE [22] using 1000 bootstrap replicates, while BI analysis was performed with MrBayes [23] running 2,000,000 generations of Markov chain Monte Carlo simulations, sampling every 1000 generations with the first 25% discarded as burn-in. The final results of the phylogenetic analysis were visualized using the Interactive Tree of Life (iTOL) online tool (https://itol.embl.de/, accessed on 1 June 2025).

### 2.6. Divergence Time Estimates Focused on Microhylidae

The BEAST v1.8.4 software was to estimate the divergence time [24]. The general time-reversible (GTR) model was chosen as it best fit our dataset, accounting for unequal base frequencies and substitution rates between nucleotide pairs. This was combined with a rigorous molecular clock to estimate the time scales. The Yule process was used as a tree prioritization. The divergence time of *M. fissipes* was estimated (4.86–10.93 MYA) based on the fossil record obtained from the TimeTree website (http://www.timetree.org, accessed on 3 June 2025). The MCMC chain was run for 20,000,000 generations, with the first 10% discarded as aging. Parameters were recorded every 2000 generations and ESS values were checked using Tracer [25], with all ESS values exceeding 300, to ensure accurate results. The tree was generated using TreeAnnotator v1.8.4 [26] and the divergence time tree was viewed and embellished using FigTree v1.4.5 (http://tree.bio.ed.ac.uk/software/figtree/, accessed on 5 June 2025).

### 2.7. Ka and Ks Analysis

To better assess selective pressure among *Microhyla* species, we calculated the Ka/Ks values for 13 protein-coding genes across 8 species [27]. A Ka/Ks ratio of 1 indicates neutral mutations, meaning mutations are not influenced by selection and are randomly fixed. A Ka/Ks ratio greater than 1 indicates positive selection, suggesting that the gene is undergoing adaptive evolution, with amino acid changes being beneficial. A Ka/Ks ratio less than 1 indicates negative (purifying) selection, indicating that the gene is conserved, with amino acid changes being harmful. The calculation results aid in analyzing the evolutionary patterns of the species and provide a better understanding of the selective pressures between gene sequences of different species [28].

## 3. Results

### 3.1. Composition of the M. fissipes Mitochondrial Genome

**Figure 1:**
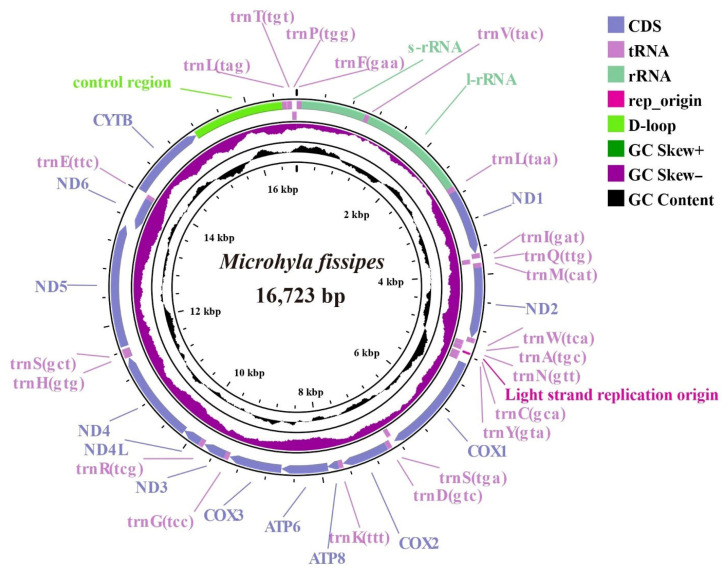
Complete mitogenome circles map of *Microhyla fissipes*; purple represents tRNA, green represents rRNA, and black areas represent GC content.

The complete mitochondrial genome of *M. fissipes* consisted of 16,723 base pairs (bp), as detailed in Table S1 (Supplementary data) and shown in Figure 1. The analysis of the *M. fissipes* mitochondrial genome revealed 38 distinct regions, including 13 protein-coding genes (PCGs), 2 ribosomal RNAs (rRNAs), 22 transfer RNAs (tRNAs), and one non-coding region (D-loop). This gene structure aligns with the typical organization observed in most animal mitochondrial genomes [29]. The base composition analysis showed the following percentages—A (28.9%), T (31.01%), C (25.51%), and G (14.58%)—with an overall A + T content of 59.91% (Table 1). Additionally, we observed a slight negative AT-skew (−0.035) and GC-skew (−0.027). Previous studies have suggested that A + T-rich regions are a major factor contributing to length variation in mitochondrial genomes, which may also apply to *M. fissipes* [30]. The light chain (L chain) of the *M. fissipes* mitochondrial genome is responsible for encoding one PCG (*ND6*) and eight tRNAs (*tRNA-Gln*, *tRNA-Ala*, *tRNA-Asn*, *tRNA-Cys*, *tRNA-Tyr*, *tRNA-Ser*, *tRNA-Glu*, and *tRNA-Pro*), while most of the genes are located on the heavy chain (H chain) (Table S1—Supplementary data). Overlapping sequences and spacer regions were found in several genes, with the largest gene overlap (17 bp) occurring between *ND5* and *ND6* and the largest spacer region occurring between *tRNA-Ser* and *ND5* (34 bp in length).

### 3.2. PCGs and Codon Usage Patterns of M. fissipes

The nucleotide composition of the 13 protein-coding genes (PCGs) in the mitochondria of *M. fissipes* was found to be analogous to that of other species of frogs. Thirteen PCGs had a total length of 11,301 bp. The AT content of the PCGs was found to be 60.45%, which is significantly higher than the 39.55% observed in the GC content. Additionally, negative values were identified for both AT skewness (−0.112) and GC skewness (−0.275), as presented in Table 1. The nucleotide biases of A and T were found to be analogous. Specifically, the first position of each codon exhibited the lowest AT content, the third position exhibited the highest AT content, and all AT-skews were positive, while AT-skews were negative in the second position. A thorough examination of the mitochondrial genome of amphibians reveals a pronounced bias in nucleotide composition, with the third codon position demonstrating the most significant deviations [31,32]. In this study, the majority of the codons terminated with either A or T. With the exception of *COX1* and *ND1*, whose start codons were ATA and GTG, respectively, all others were ATG, which were triggered by GTG codons. With regard to the termination codons, *ND1*, *ND3*, *ND4*, *ATP6*, *COX2*, and *COX3* exhibit incomplete termination. The distinguishing feature is that, among them, the termination codon of the *ND2* gene is TAG, the termination codon of the *ND5* and *COX1* genes is AGG, the termination codon of *ND6* is AGA, and the termination codon of the remaining genes is TAA.

**Figure 2:**
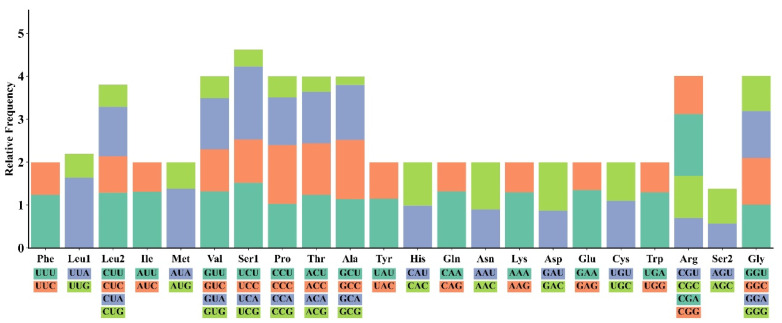
Relative Synonymous Codon Usage (RSCU) in *Microhyla fissipes* mitogenome.

**Figure 3:**
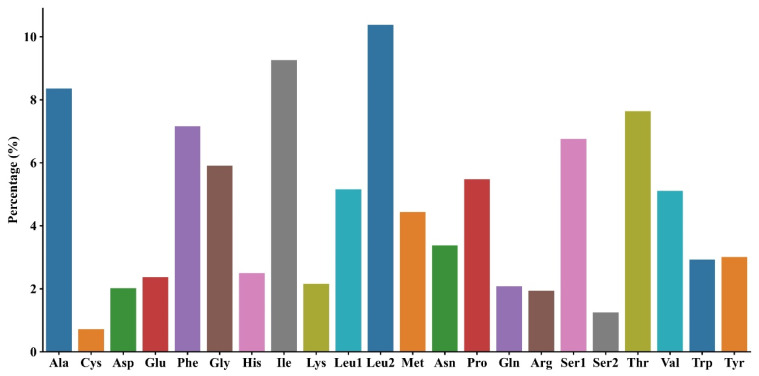
Amino acid composition of protein-coding genes in *Microhyla fissipes*.

A total of 5574 codons were identified in the 13 PCGs of *M. fissipes*. The relative values of codon usage are demonstrated in Figure 2. It is noteworthy that AUU (isoleucine; Ile), UUU (phenylalanine; Phe), and UUA (leucine; Leu) were identified as the most commonly used codons. The least frequently used codons were GCG (alanine; Ala), CGU (arginine; Arg), and GUG (valine; Val). The AT bias in the *M. fissipes* mitochondrial genome was found to influence codon usage patterns. Our analysis identified overlapping gene arrangements in three pairs of protein-coding genes: *ATP8* and *ATP6*, *ND4L* and *ND4* on the same strand, and *ND5* and *ND6* on opposite strands. As illustrated in Figure 3, among the amino acids identified in *M. fissipes*, leucine (L) demonstrates the highest percentage (15.54%), exhibiting its presence across six distinct codons, classified as L1 and L2. Conversely, cysteine (C) exhibits the lowest percentage among the identified amino acids, with a representation of 0.72%. The results obtained from the study indicated that the T and A bases were more prevalent than the G and C bases. This finding suggests a significant preference for A or T at the third codon position, indicating a bias in the genetic code.

### 3.3. Transfer RNA and Ribosomal RNA Genes

A total of 22 tRNAs were identified in the mitogenome of *M. fissipes*, with the leucine and serine genes represented twice (Table S1—Supplementary data). The secondary structures of all tRNAs are shown in Figure S1, with sequence lengths ranging from 66 to 73 base pairs. Analysis of the predicted secondary structures revealed that trnS1 lacked the dihydrouracil (DHU) loop and therefore could not form a complete cloverleaf conformation. However, the remaining tRNAs were able to form the standard cloverleaf secondary structure. We found that the tRNA of *M. fissipes* is essentially characterized by the traditional A-U and G-C base pairing in the secondary structure of mitochondrial tRNAs, but it was also found to contain a number of mismatched base pairs, e.g., U-U, A-A, and A-C.

**Figure 4:**
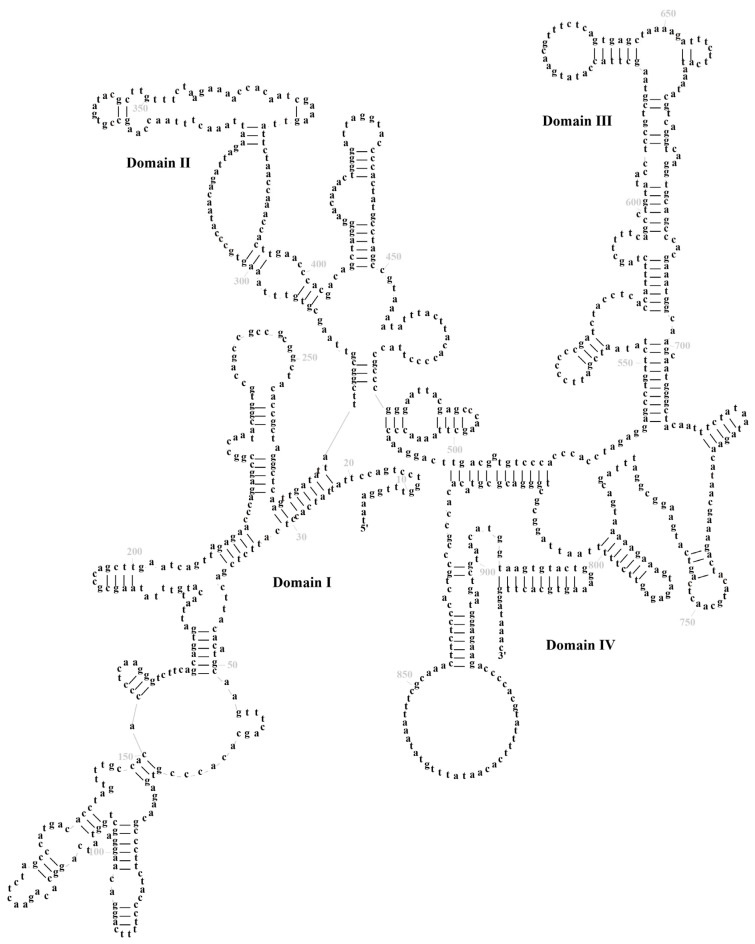
The prognostic map of *12S rRNA* secondary structures in *Microhyla fissipes*.

**Figure 5:**
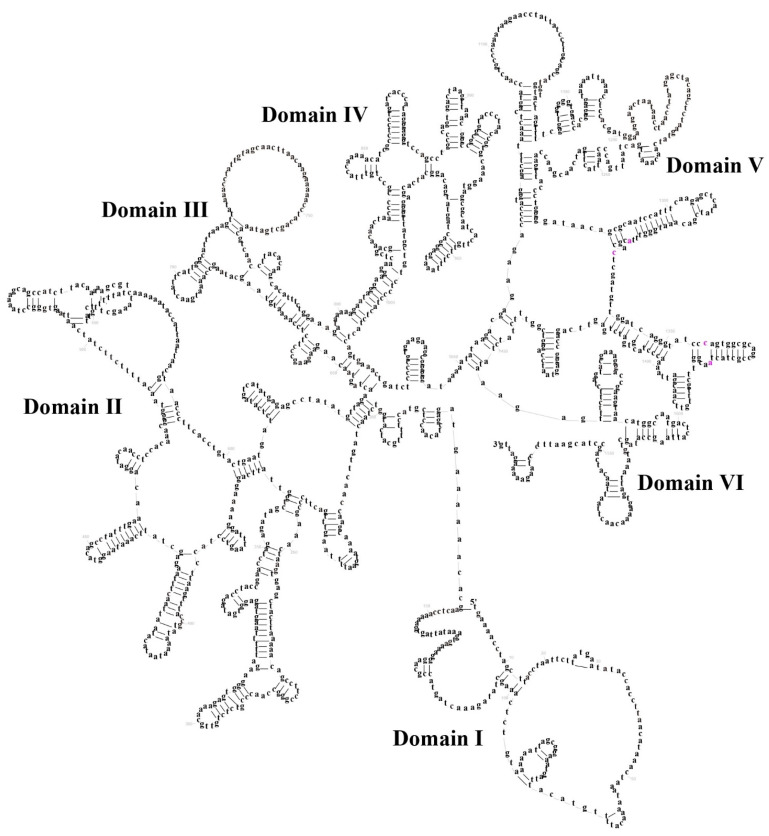
The prognostic map of *16S rRNA* secondary structures in *Microhyla fissipes*.

In *M. fissipes*, the total length of ribosomal RNA (rRNA) was determined to be 2516 base pairs (bp), with an A + T content of 58.62%. The *12S rRNA* gene was found to span 937 bp, its position demarcated by the *tRNA-Phe* and *tRNA-Val*. The *16S rRNA* gene was observed to be 1579 bp in length, situated between the *tRNA-Val* and *tRNA-Leu* (Table S1—Supplementary data). *12S* and *16S rRNAs* are located between *tRNA-Phe* and *tRNA-Leu* and separated by *tRNA-Val*, a feature that is common to the vertebrate mitogenome [33]. The predicted *12S rRNA* and *16S rRNA* gene secondary structures were constructed separately. The secondary structure of *12S rRNA* comprises 40 stem–loop structures, encompassing domains I–IV. Domains I and II are classified as variable regions, while domains III and IV are designated as conserved regions (Figure 4). The secondary structure of *16S rRNA* comprises 71 stem–loop structures, which form six structural domains (III–VI) (Figure 5). These domains are distinguished from one another by a single-stranded segment. Of particular note are structural domains I–III and VI, which exhibit variability, and structural domains IV and V, which demonstrate conservation. The initiation of L-strand replication (*O_L_*) with a 31-base pair (bp) segment between *tRNA-Asn* and *tRNA-Cys* may result in the formation of a stem–loop secondary structure, exhibiting characteristics analogous to most vertebrate motifs [29,31,34]. This motif has been demonstrated to be associated with the transition from RNA to DNA synthesis in humans [35].

### 3.4. The Control Region

A thorough investigation was conducted to ascertain the presence of conserved structural elements within the control region of *M. fissipes.* The largest non-coding region in the mitochondrial genome is the control region (*CR*, 1339 bp), with an AT content of 61.09%, located between *Cytb* and *tRNA-Leu* (Table S1—Supplementary data). Furthermore, the *CR* model demonstrates negative AT skew (−0.083) and GC skew (−0.278) in *M. fissipes*.

### 3.5. Synonymous and Nonsynonymous Substitution Rate

**Figure 6:**
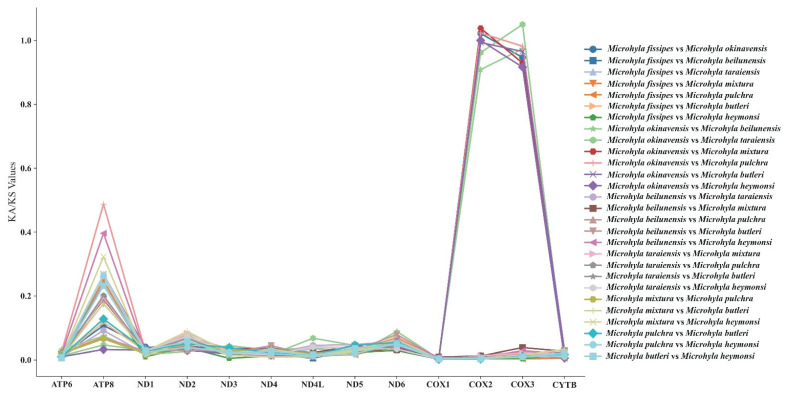
The Ka/Ks values among the *Microhyla* species.

In order to assess the selection pressure on mitochondrial protein-coding genes, we analyzed the rate of non-synonymous (Ka) and synonymous (Ks) substitutions in 13 protein-coding genes between eight species (*M. fissipes*, *M. okinavensis*, *M. beilunensis*, *M. taraiensis*, *M. mixtura*, *M. pulchra*, *M. butleri and M. heymonsi*). An analysis of the data revealed that the majority of the genes exhibited Ka/Ks ratios less than 1 (Figure 6). This finding indicates that most of the genes undergo a strong purifying selection effect. In the *COX2* and *COX3* genes, the Ka/Ks ratio is greater than 1 between some species, while the remaining PCGs show purifying selection, indicating that the evolutionary process aims to eliminate harmful mutations. It is noteworthy that the Ka/Ks ratios of the *Cytb*, *COX1*, and *ATP6* genes were marginally lower compared to the other genes. This finding suggests that these genes may exhibit a more conserved function in these regions. The results of this study contribute to the scientific community’s understanding of the evolutionary dynamics of the mitochondrial genome in the family Microhylidae. Furthermore, they have systematic implications for resolving phylogenetic relationships within the subfamily.

### 3.6. Phylogenetic Analyses

**Figure 7:**
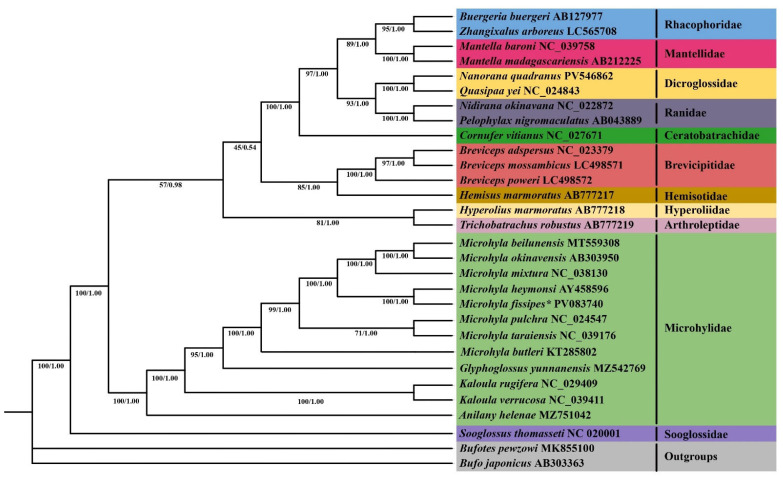
Maximum-likelihood (ML) and Bayesian inference (BI) phylogenetic tree of sixteen frog species based on 13 protein-coding genes. ML bootstraps and BI posterior probabilities are shown at the node. The GenBank accession numbers are listed following species names. Species sequenced in this study are indicated by Latin names with asterisks.

Phylogenetic trees were reconstructed based on 13 protein-coding genes from 30 species, using *Bufotes pewzowi* and *Bufo japonicus* as outgroups. Both maximum likelihood (ML) and Bayesian inference (BI) analyses produced identical topologies of species lineages (Figure 7). The results demonstrate that the phylogenetic relationships within Microhylidae—encompassing *Kaloula*, *Microhyla*, *Glyphoglossus*, and *Anilany*—are consistent with those reported in previous classification systems [6]. Among them, *Microhyla* and *Glyphoglossus* formed a sister group with a guide value of 95, indicating that they are more closely related genetically. Within the target lineage *Microhyla*, both ML and BI trees showed the following arrangement: (*Microhyla butleri*, ((*Microhyla pulchra*, *Microhyla taraiensis*), ((*Microhyla fissipes*, *Microhyla heymonsi*), (*Microhyla mixtura*, (*Microhyla okinavensis*, *Microhyla beilunensis*))))) (Figure 7). The study revealed that *M. fissipes* is sister to the branch comprising *M. heymonsi*, *M. beilunensis*, *M. okinavensis*, and *M. mixtura*. This finding is consistent with previous studies [1].

### 3.7. Divergence Time Estimation of Microhylidae Species

**Figure 8:**
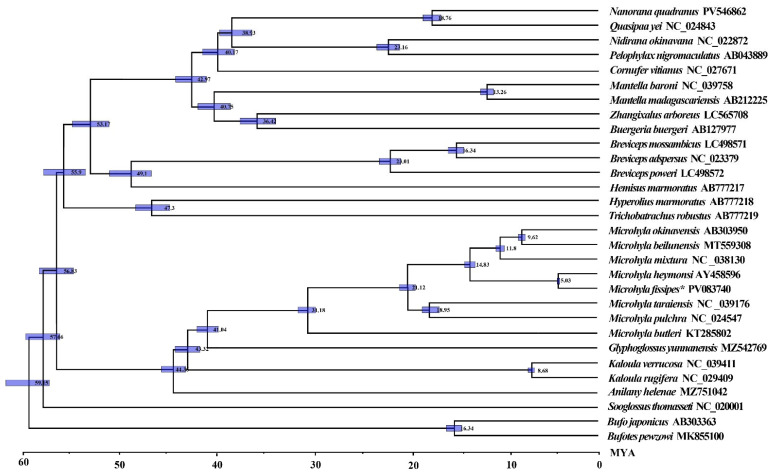
The divergence time of Microhyloidea with 95% highest posterior probability density. Numbers nearby nodes refer to divergence times. Blue bars indicate 95% highest posterior density intervals (HPD) for nodes of interest. The “*” represents the current study.

Divergence times among Microhylidae were estimated using Bayesian methods implemented in BEAST. The divergence of Microhyloidea occurred during the Eocene period of the Paleocene Epoch, approximately 56.83 Mya. Subsequently, it underwent a bifurcation into two main branches, one of which gave rise to families such as Rhacophoridae, Mantellidae, and Dicroglossidae (Figure 8). The divergence of *M. fissipes* and *M. heymonsi* is estimated to have occurred during the Neogene period of the Miocene epoch, approximately 5.03 Mya, while the origin of *Microhyla* can be traced back to the Eocene epoch, approximately 41.04 Mya.

The results of the present study suggest that Microhylidae originated in the Paleocene, with divergence among genera occurring afterward. During the Oligocene, this group underwent rapid diversification and dispersal, a phenomenon strongly shaped by concurrent geological and climatic shifts. The Paleocene (23–66 Mya) was characterized by an overall transition from warm to cool conditions, with intermittent temperature fluctuations that included an initial modest warming trend. Climatic temperatures reached their zenith during the early Eocene (33.7–53 Mya), followed by a pronounced cooling phase in the late Eocene. Throughout the middle and late Paleocene, the temperature decline was mainly due to increased global ice volume, sea level fall, expansion of grasslands, and reduction in vegetation cover, including tropical broadleaf forests [36]. The diversification of Microhylidae and its intragroup morphological disparity were strongly influenced by contemporaneous geological dynamics and the biogeographic patterns of their host plants during this epoch.

## 4. Discussion

### 4.1. Structural and Compositional Features of the M. fissipes Mitochondrial Genome

The mitogenome of *M. fissipes* exhibits a structure consistent with most vertebrates [8,29], characterized by a pronounced AT bias (59.91%)—a hallmark feature of vertebrate mitochondrial genomes likely influenced by selection related to replication and transcription efficiency [37]. This compositional tendency is further supported by negative AT-skew and GC-skew values, reflecting a nucleotide pattern where guanine (G) is the least abundant, a trend commonly observed in amphibians [38,39]. Such biases may contribute to mutation patterns and functional constraints within mitochondrial genes. The gene order and transcriptional direction are consistent with those reported in *M. taraiensis* [10]. The control region (CR) was confirmed to harbor key regulatory signals for replication and transcription initiation [40]. Additionally, as typical among amphibians, the total mitogenome length shows interspecific variation, which is also evident in *M. fissipes* [41].

A key feature observed in several protein-coding genes (*ND1*, *ATP6*, *COX2*, *COX3*, *ND3*, and *ND4*) is the presence of incomplete termination codons (T--), which are presumably converted to functional TAA stop codons via post-transcriptional polyadenylation [42]. Additionally, overlapping reading frames were detected in three gene pairs: *ATP8-ATP6* (10 bp), *ND4L-ND4* (7 bp), and *ND5-ND6* (17 bp, opposite strand), along with shared nucleotides between certain protein-coding and tRNA genes. These compact genomic arrangements highlight the evolutionary pressure to minimize mtDNA size while maintaining functional integrity, a phenomenon widely documented across metazoan mitochondrial genomes [43]. The retention of these structural and compositional traits in *M. fissipes* underscores their critical role in mitochondrial function and evolutionary adaptation. Due to the lack of a dihydrouracil (DHU) ring in *trnS1* in the secondary structure of tRNA, it cannot form a standard cloverleaf structure. As noted by Ohtsuki et al. [44], the lack of the DHU arm in *tRNA-Ser* suggests that incomplete tRNAs can still be adapted to the ribosome by adjusting their functional and structural conformation. And stem mismatches seem to be a common phenomenon in mitochondrial tRNA genes and may be repaired by a post-transcriptional editing process [45].

### 4.2. Phylogenetic Analysis of Microhylidae

Over the past decade, molecular assessments have revolutionized our understanding of amphibian diversity in tropical island ecosystems, consistently revealing substantial underestimations of species richness [46,47]. This pattern is particularly evident in morphologically conserved groups such as microhylid frogs, where pronounced genetic differentiation frequently occurs despite minimal morphological variation at the species level [48]. The genus *Microhyla* exemplifies this trend, with accumulating molecular evidence from multiple studies [49,50] demonstrating that current taxonomic frameworks significantly underestimate its true diversity. These molecular findings, which reveal a severe underestimation of species diversity, have urgent practical implications for the conservation of amphibians in tropical island ecosystems. Unidentified cryptic species cannot receive targeted conservation assessments and actions, and are at high risk of extinction. Specifically regarding the *M. fissipes* complex, our research suggests it may contain multiple deeply divergent cryptic species. This implies that the current understanding based on a single-species concept severely misleads conservation practices: the actual distribution range of each cryptic species may be far smaller than recorded, population sizes are significantly overestimated, and the unique threats they face cannot be accurately assessed. This situation is particularly concerning on tropical islands with fragile and highly fragmented habitats. There is an urgent need for comprehensive taxonomic revisions based on molecular evidence, followed by independent conservation assessments for each newly identified species and the development of tailored management strategies. This study contributes to this growing body of knowledge, providing important taxonomic insights into this morphologically complex yet genetically diverse amphibian group.

Recent molecular phylogenetic studies on *Microhyla* frogs have yielded both congruent and conflicting topologies depending on the genetic markers employed. Our analysis of 13 protein-coding genes, 2 rRNAs, and 22 tRNAs recovered a lineage structured as (*Microhyla butleri*, ((*Microhyla pulchra*, *Microhyla taraiensis*), ((*Microhyla fissipes*, *Microhyla heymonsi*), (*Microhyla mixtura*, (*Microhyla okinavensis*, *Microhyla beilunensis*))))), consistent with earlier findings by Kurabayashi et al. [51], jiang et al. [8] and Yong et al. [52], but differing from an alternative topology proposed by Matsui et al. [53]. However, other studies using different molecular datasets have generated discordant results: analyses of *12S rRNA*, *BDNF*, tyrosinase, and 28S sequences placed *M. butleri* distant from *M. ornata* [54], while combined mitochondrial and nuclear gene datasets [55] and *12S rRNA* sequences [56] supported distinct placements of *M. butleri* relative to other species. Our maximum likelihood and Bayesian analyses strongly support *M. butleri* in a basal position, corroborating van der Meijden et al. [57], Pyron & Wiens [55], and Howlader et al. [56], while also identifying *M. fissipes* as sister to *M. okinavensis* + *M. mixtura* + *M. beilunensis* and *Glyphoglossus yunnanensis* as sister to *Microhyla*—findings that align with previous microhylid phylogenies [51]. These results collectively highlight both consistent patterns and persistent conflicts in *Microhyla* systematics, underscoring the need for integrative taxonomic approaches to resolve these evolutionary relationships.

### 4.3. Divergence Time and Selective Pressure

The BEAST-derived chronogram (Figure 8) indicates that the most recent common ancestor (MRCA) of *Microhyla* and *Glyphoglossus* emerged during the late Paleocene to early Eocene (ca. 41.0 Mya, 95% HPD: 38.1–44.2). This estimate aligns with Feng et al. [58] (48.8 Mya, 45.9–53.2) and Gorin et al. [7] (50.8 Mya, 44.1–57.0) but predates Garg & Biju’s [59] proposed origin at 61.5 Mya (56.6–66.5). The *Microhyla* + *Glyphoglossus* clade underwent rapid radiation in the middle Eocene (43.8 Mya, 38.7–49.1), slightly more recent than Garg & Biju’s [59] estimation of 48.7 Mya (44.1–53.2) for this cladogenetic event. Subsequent diversification within these genus-level endemic lineages commenced in the early to mid-Oligocene (20–35 Mya), consistent with previous findings. Detailed node ages with corresponding 95% highest posterior density (HPD) intervals are provided in Figure 8.

Our results suggest that Microhylidae diverged around 56.83 Mya, consistent with published estimates [60]. The early Oligocene (~34 Mya) marked a critical period for Microhylidae diversification, coinciding with global climatic shifts following Antarctic glaciation [61]. These environmental changes, characterized by cooling and aridification, likely promoted allopatric speciation through habitat fragmentation while creating new ecological opportunities for amphibian radiation [62]. Such paleoclimatic conditions may explain both the timing and pattern of Microhylidae diversification, including the evolutionary trajectory of *M. fissipes* and closely related species. While mitochondrial phylogenies provide valuable insights, comprehensive understanding of Microhylidae evolution will require integration of nuclear genomic data with expanded taxonomic sampling and morphological evidence.

Analysis of selective pressures reveals distinct evolutionary patterns among mitochondrial protein-coding genes (PCGs) in *M. fissipes*. The majority of genes exhibit strong purifying selection (ω = ka/ks << 1), consistent with their essential metabolic functions [63,64]. Notably, *COX1* demonstrates exceptional evolutionary constraint (ω as low as 0.0018), reflecting its critical role in oxygen reduction within the electron transport chain [65]. Such extreme conservation aligns with mitochondrial genes’ fundamental position in cellular energy production [66], where structural integrity must be maintained to preserve core metabolic functions. A striking exception emerges in *ATP8*, which shows significantly elevated ω values (0.2–0.4876) compared to other PCGs. This pattern suggests either: (1) reduced functional constraints due to its minor contribution to ATP synthase complex activity [67], or (2) positive selection at specific sites responding to environmental adaptations [68]. The observed pattern in *M. fissipes* corroborates widespread reports of relaxed selection on *ATP8* across taxa [67], warranting future investigation through site-specific selection analyses to elucidate its evolutionary drivers.

Unlike other comparisons, the *COX2* and *COX3* genes in *M. fissipes* and *M. okinavensis* exhibit unusually high Ka/Ks ratios (approaching or slightly exceeding 1), significantly deviating from the expected pattern of strong purifying selection typically observed in mitochondrial core genes. This anomalous signal suggests that the relevant genes may have undergone a unique evolutionary trajectory: a temporary relaxation of functional constraints [69] (e.g., ecological niche shifts reducing selective pressure) or weak positive selection events [68] (e.g., subunits fine-tuned to adapt to specialized environments) may have driven the recent evolution of these key genes in the *COX* complex of this lineage. Notably, such high Ka/Ks values are observed exclusively in the *M. okinavensis*-related pair, indicating lineage-specific evolutionary dynamics [70]; while in other interspecific comparisons, *COX2/COX3* strictly follow a purifying selection pattern (Ka/Ks << 1). Future studies should combine site-specific selection models with functional experiments to verify whether these mutations drove adaptive differentiation in the *M. okinavensis* lineage or reflect compensatory mechanisms in mitochondrial-nuclear coevolution.

## 5. Conclusions

Here, we characterized the complete mitochondrial genome of *M. fissipes*. Comparative genomic analysis demonstrated high conservation across Microhylidae species, evidenced by stable base composition, uniform genome size and organization, conserved protein-coding genes (PCGs) with consistent codon usage, and structurally conserved tRNA secondary folds. To elucidate phylogenetic relationships, we performed Bayesian inference (BI) and maximum likelihood (ML) analyses using 13 PCGs. The resulting phylogeny exhibited a well-resolved topology with robust nodal support across all branches. Both ML and BI methods yielded congruent tree topologies, strongly confirming the monophyly of recognized genera within the group. Our findings further clarify the phylogenetic relationships within Microhylidae, strongly supporting the clade (((*Glyphoglossus*, *Microhyla*), *Kaloula*), *Anilany*). Within *Microhyla*, both ML and BI analyses consistently recovered the following topology: (*Microhyla butleri*, ((*Microhyla pulchra*, *Microhyla taraiensis*), ((*Microhyla fissipes*, *Microhyla heymonsi*), (*Microhyla mixtura*, (*Microhyla okinavensis*, *Microhyla beilunensis*))))). Divergence time estimation places the origin of Microhylidae near the Paleocene-Eocene boundary (Paleogene period), providing key temporal context for the group’s evolutionary history. These results deliver essential phylogenetic insights, establishing a robust framework for future studies on Microhylidae systematics and evolution. The well-resolved relationships and divergence timing presented here will facilitate deeper investigations into the family’s diversification patterns and biogeographic history. This study emphasizes that accurate classification and delineation are the cornerstones of effective protection for morphologically protected amphibians, and *M. fissipes* serves as a model for incorporating molecular systematics into wildlife management frameworks.

## Figures and Tables

**Table 1:** Nucleotide composition of different partitions from *Microhyla fissipes* mitogenomes.

Region	Size (bp)	A (%)	T (%)	G (%)	C (%)	A + T (%)	G + C (%)	AT Skew	GC Skew
Mitogenome	16,723	28.9	31.01	14.58	25.51	59.91	40.09	−0.035	−0.272
tRNAs	1534	28.68	28.29	22.82	20.21	56.98	43.02	0.007	0.061
rRNAs	2516	33.47	25.16	18.36	23.01	58.62	41.38	0.142	−0.112
PCGs	11,301	26.83	33.63	14.34	25.21	60.45	39.55	−0.112	−0.275
PCGs (1st)	3765	28.15	25.79	23.72	22.34	53.94	46.06	0.044	0.030
PCGs (2nd)	3765	17.58	41.43	12.8	28.18	59.02	40.98	−0.404	−0.375
PCGs (3rd)	3765	34.79	33.55	6.51	25.15	68.34	31.66	0.018	−0.589
CR	1339	28.01	33.08	14.04	24.87	61.09	38.91	−0.083	−0.278

## Data Availability

Mitochondrial genome sequence data supporting the findings of this study are openly available from GenBank of the National Center for Biotechnology Information (NCBI) at https://www.ncbi.nlm.nih.gov (accession number: PV083740).

## Supplementary Materials

The following supporting information can be downloaded at: https://www.mdpi.com/article/10.3390/biology14101342/s1. Figure S1: Secondary structure predictions for 22 tRNA genes of *Microhyla fissipes*; Table S1: Annotation of the mitochondrial genome of *Microhyla fissipes*.
