# Supplementary material for: Mitogenomic Characterization of Microhyla fissipes and Its Implications for Phylogenetic Analysis in Microhylidae
Source: Biology (Basel). 2025 Oct 1;14(10):1342. doi: 10.3390/biology14101342 (PMC12562188; doi:10.3390/biology14101342)
Supplement: Supplementary file 1 [file Figure S1. Secondary structure predictions for 22 tRNA genes of Microhyla fissipes..pdf › Figure S1. Secondary structure predictions for 22 tRNA genes of Microhyla fissipes..pdf]

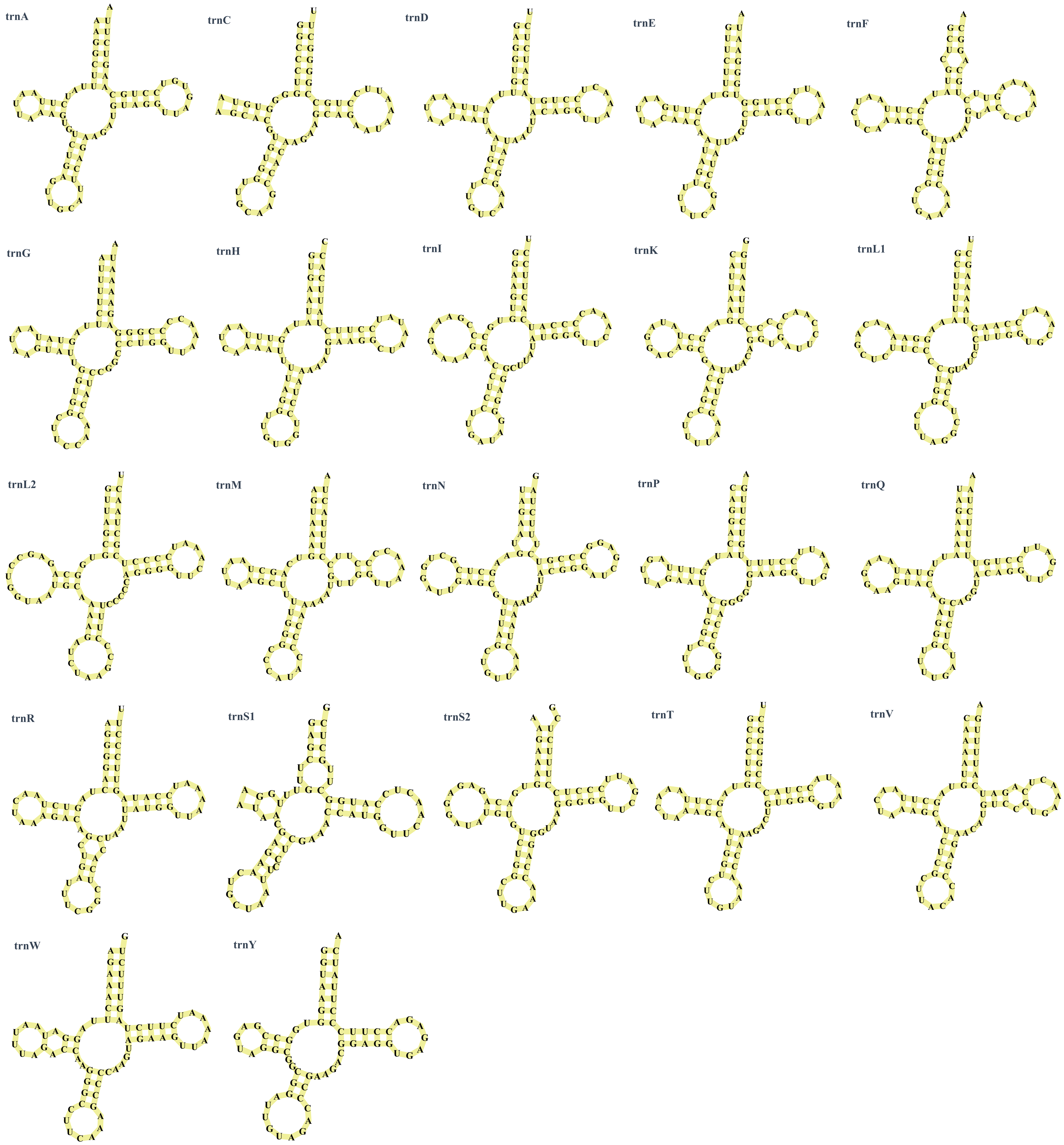
trnA
trnC
trnD
trnE
trnF
trnG
trnH
trnI
trnK
trnL1
trnL2
trnM
trnN
trnP
trnQ
trnR
trnS1
trnS2
trnT
trnV
trnW
trnY
